# Surface Electric Fields Increase Human Osteoclast Resorption through Improved Wettability on Carbonate-Incorporated Apatite

**DOI:** 10.1021/acsami.1c14358

**Published:** 2021-12-03

**Authors:** Leire Bergara-Muguruza, Keijo Mäkelä, Tommi Yrjälä, Jukka Salonen, Kimihiro Yamashita, Miho Nakamura

**Affiliations:** † Medicity Research Laboratory, Faculty of Medicine, University of Turku, Tykistökatu 6, 20520 Turku, Finland; ‡ Turku University Hospital, University of Turku, Luolavuorentie 2, 20700 Turku, Finland; § Department of Anesthesia and Intensive Care, University of Turku, Luolavuorentie 2, 20700 Turku, Finland; ∥ Graduate School of Medical and Dental Science, Tokyo Medical and Dental University, 1-5-45 Yushima, Bunkyo-ku, Tokyo 113-8510, Japan; ⊥ Institute of Biomaterials and Bioengineering, Tokyo Medical and Dental University, 2-3-10 Kanda-Surugadai, Chiyoda, Tokyo 1010062 Japan; # Graduate School of Engineering, Tohoku University, 6-6 Aramaki Aza Aoba, Aoba-ku, Sendai, Miyagi 9808579 Japan

**Keywords:** wettability, surface free energy, osteoclast, carbonate-incorporated apatite, electrical polarization

## Abstract

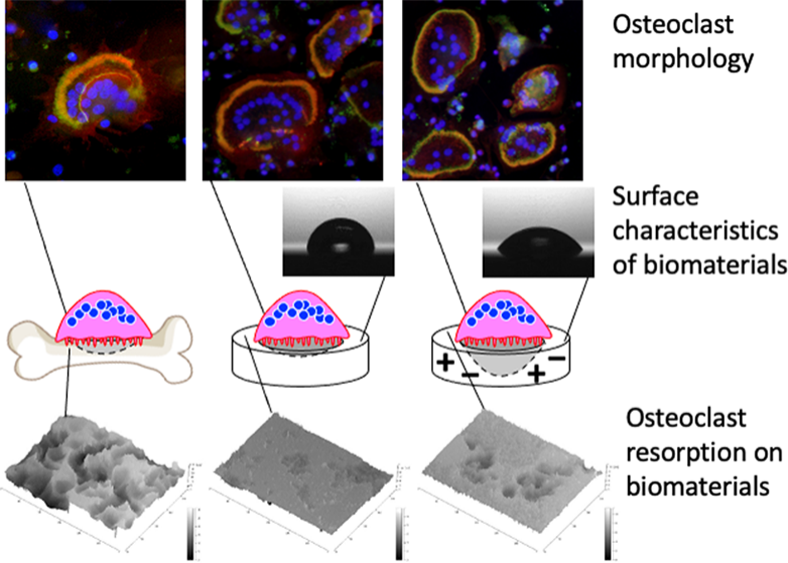

Osteoclast-mediated bioresorption can be an efficient means of incorporating the dissolution of biomaterials in the bone remodeling process. Because of the compositionally and structurally close resemblance of biomaterials with the natural mineral phases of the bone matrix, synthetic carbonate-substituted apatite (CA) is considered as an ideal biomaterial for clinical use. The present study therefore investigated the effects of electrical polarization on the surface characteristics and interactions with human osteoclasts of hydroxyapatite (HA) and CA. Electrical polarization was found to improve the surface wettability of these materials by increasing the surface free energy, and this effect was maintained for 1 month. Analyses of human osteoclast cultures established that CA subjected to a polarization treatment enhanced osteoclast resorption but did not affect the early differentiation phase or the adherent morphology of the osteoclasts as evaluated by staining. These data suggest that the surface characteristics of the CA promoted osteoclast resorption. The results of this work are expected to contribute to the future design of cell-mediated bioresorbable biomaterials capable of resorption by osteoclasts and of serving as a scaffold for bone regeneration.

## Introduction

Cell-mediated bioresorption is a biological process in which biomaterials are resorbed by cells and thereby either partially or completely disappear from implantation sites over a period of time.^1^ In the case of an ideal bioresorbable biomaterial intended for bone regeneration, no foreign material will remain after bone restoration and the load-bearing capacity at the restored site will be similar to that of the natural bone tissue.^1^ Hydroxyapatite (HA) and its analogue β-tricalcium phosphate (β-TCP) have excellent biocompatibility and osteoconductivity, both of which are required for orthopedic biomaterials and so are frequently used in clinical work. HA is a poorly resorbable biomaterial while β-TCP is soluble in vivo. The degradation of β-TCP thus proceeds via solution-mediated chemical dissolution, such that this material will dissolve under physiological conditions. Cell-mediated bioresorption is a new technique that is advantageous because it allows for the dissolution of biomaterials after the bone remodeling process in conjunction with bone resorption and formation. Because osteoclasts are responsible for bone resorption, the development of osteoclast-mediated bioresorbable biomaterials is imperative for bone regeneration in vivo. Interestingly, the incorporation of carbonate ions within the HA crystal structure has been experimentally validated to increase osteoclast differentiation and resorption,^2,3^ even though stoichiometric HA cannot be resorbed by osteoclasts. Carbonate-substituted apatite (CA), in which 2 to 8 wt % of the material is substituted by carbonate ions and which also has low concentrations of potassium, magnesium, chloride, and sodium in its crystal structure, resembles the natural mineral phases in bone matrices.^4^ As a result of the similar compositions of the mineral phases in CA and bones, the former is considered to be an ideal clinical biomaterial for bone remodeling.

Osteoconduction at the so-called interface between an implanted biomaterial and the bone tissue proceeds via six stages: protein adsorption, osteoblast attachment, proliferation, differentiation, formation of mineralized extracellular matrix, and bone remodeling.^5^ In the final stage, the osteoclasts play an invaluable reconstructive role by resorbing the immature bone matrix formed by osteoblasts and bioresorbable materials. For this reason, the ability of osteoclasts to resorb HA and CA surfaces and to perform additional remodeling to reconstruct the immature bone matrix formed by osteoblasts into mineralized bone has to be assessed. Even so, although the importance of the interactions between osteoclasts and biomaterials is widely acknowledged, these interactions are not yet fully understood.

There have been many studies evaluating the interfaces between biomaterials and cells, with the aim of controlling cellular behaviors such as adhesion, proliferation, and differentiation. Studies of osteoclasts cultured on inorganic biomaterials, such as β-TCP, HA, and calcium carbonate, have been used to evaluate osteoclast activity in vitro. Consequently, it has been determined that in vitro osteoclast resorption is affected by the type of inorganic biomaterials involved,^6−8^ the incorporation of ions (such as carbonate^9,10^ and silicon^11^), the surface energy,^2^ the physicochemical dissolution process,^12^ the surface roughness of the substrate,^13,14^ and the surface crystallinity.^15,16^

Human osteoclasts adopt a spread cell morphology on A-type CA, in which carbonate ions are substituted at hydroxide sites in the HA crystal structure. This process, which can be demonstrated by actin staining, is caused by the decreased surface energy compared with the values for HA and calcium carbonate.^2^ A previous work has reported that rabbit osteoclasts resorb CA but not HA because CA exhibits significant physicochemical dissolution under acidic conditions.^12^ The resorption of CA by human osteoclasts has also been observed in conjunction with AB-type CA crystals based on HA incorporation over 2.4 wt % carbonate,^10^ and mouse osteoclasts resorbed B-type CA with 7.7 wt % carbonate.^17^ AB-type CA, in which carbonate ions are substituted at hydroxide and phosphate sites in the HA crystal structure, have been found to be resorbed by human osteoclasts along with the subsequent acceleration of the proliferation of human osteoblast-like cells and collagen synthesis.^9^ The increased osteoblast proliferation and differentiation associated with osteoclast resorption suggest that CA affects new bone formation through the activities of osteoclasts.

Recently, we demonstrated that electrically polarized HA enhances early-stage protein adsorption after in vivo implantation,^18^ as well as the initial adhesion and migration of osteoblast-like cells in vitro^19^ and osteoconductivity in vivo,^20^ compared with standard HA. Two of the most important factors related to the enhancement of biological reactions through electrical polarization are the attendant increase in the surface free energy and improved surface wettability of the solid biomaterial.^21,22^ Other research groups have reported similar effects of electrical polarization on osteoblast-like cells, including greater wettability.^23,24^ Although the promotion of the initial stages of osteoconduction by electrical polarization has been reported, the effects on osteoclast behavior have not yet been elucidated. We are especially intrigued by the interactions between osteoclasts and polarized biomaterials, which might help to clarify the mechanism by which polarization-induced effects occur in osteoconduction. In the present study, we therefore combined approaches from biology and materials science and used HA and CA with charged surfaces induced by polarization to better understand the interactions between osteoclasts and biomaterials.

## Materials and Methods

### Preparation of Biomaterials

The wet chemical method was applied to the synthesis of starting HA powders from analytical grade reagents calcium hydroxide and phosphoric acid using a wet method.^3^ The dried precipitates after water-rinsing were calcined at 850 °C to obtain HA precursors and thereafter pressed in a mold at 200 MPa. The HA compacts were then subjected to sintering at 1250 °C for 2 h in a saturated water vapor steam. The precursor powders of CA were synthesized using a modified procedure established by Doi et al.^3,12^ The analytical grade powders of sodium carbonate, disodium hydrogen phosphate, and calcium nitrate tetrahydrate were wet chemically mixed with a CO_3_/PO_4_ molar ratio of 5.^3,12^ The compacts of rinsed and dried powders were pressed in a mold at 200 MPa and subjected to sintering for 2 h in a carbon dioxide atmosphere at 780 °C to obtain dense CA bodies without carbonate loss for surfaces. Bone slices were also prepared as control samples for osteoclast culture trials, using a procedure previously described in the literature.^3^ In brief, a frozen cortical bone derived from the bovine femur was cut into slices with a 130–180 μm by a diamond saw (Buehler, Lake Bluff, IL, USA), after which the bone slices were ultrasonically cleaned in distilled water.

Both kinds of the biomaterial specimens were polished down to a thicknesses of 0.8 mm by polishing of 5 μm in grain size. After polishing, the HA and CA specimens with ϕ7 mm in a diameter were washed in ethanol with ultrasonication. The surface roughness of each specimen was measured with a color laser microscope (Olympus, OLS4100, Tokyo, Japan) and the specimens with an apparent density of more than 98% and an Ra value of 0.24 ± 0.05 μm were used for further experimentation.

The electrical polarization of the HA and CA specimens were undertaken due to the same method in our previous work,^21^ those specimens, sandwiched with a pair of platinum plate electrodes, were subjected to direct current electric fields of 5 and 2 kV/cm at 400 and 350 °C for 1 and 0.5 h, respectively. Because the electrical polarization provides negatively and positively charged surfaces, the negatively charged HA and CA surfaces are denoted herein as HA-N and CA-N, respectively, while the positively charged ones are referred to as HA-P and CA-P, respectively.

Thermally stimulated depolarization current (TSDC) measurements were performed to confirm polarization of the HA and CA specimens in air from room temperature (RT) to 800 °C with a heating rate of 5.0 °C/min according to a method described in our previous work.^22^ The depolarization current was determined with a Hewlett-Packard 4140B pA meter. The values of stored electric charges (*Q*) were calculated by the mathematical integration of the TSDC spectra according to the equation
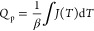
1where *J*(T) and β are the observed dissipation current density at temperature *T* and the heating rate, respectively. The values of *Q*, an activation energies (*E*_a_), and a relaxation half-life period (τ) values associated with depolarization were obtained from the TSDC data using the equations, respectively
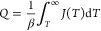
2and
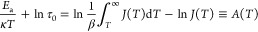
3

The value of τ, the relaxation process at 37 °C, was calculated according to the Arrhenius law
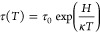
4where τ_0_ and κ are a pre-exponential factor and the Boltzmann constant.

### Structural and Surface Characterization

Phase identification by X-ray diffraction (XRD) analyses was performed on HA and CA samples with and without the polarization treatment to assess an effect of polarization on structural change, under the conditions of Cu Kα radiation with 40 kV and 40 mA (Philips PW1700). Spectroscopic analyses on local structures were also done by attenuated total reflectance–FTIR spectroscopy (ATR–FTIR) with each polarized HA and CA sample at five different points (PerkinElmer spectrum BX spotlight spectrophotometer with a diamond ATR attachment). Prior to the ATR–FTIR analysis, the specimens were held in air at 20 ± 2 °C for 48 h in a desiccator to maintain the same atmosphere as that of the FTIR equipment. Scanning was conducted from 4000 to 400 cm^–1^, with 64 scans averaged for each spectrum.

The effects of polarization on surface properties were conducted both on HA and CA by the evaluation of surface free energy values, determined with the contact angle measurements using a dual liquid phase method. Specifically, the contact angles (°) of water on the HA and CA surfaces were measured in hydrocarbon oils with various surface energies such as hexane (18.4 mJ/m^2^), heptane (20.1 mJ/m^2^), octane (21.7 mJ/m^2^), decane (23.8 mJ/m^2^), and hexadecane (27.5 mJ/m^2^). The surface free energies were then calculated according to Jouany’s equation
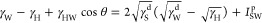
5where subscripts W, H, and S correspond to water, hydrocarbon, and solid, respectively, γ_S_^d^ and γ_W_^d^ are the dispersion components, and I_SW_^p^ is the nondispersive interaction between the solid and water as expressed by
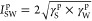
6where γ_S_^p^ and γ_W_^p^ are the polar (nondispersive) components.

According to Fowkes, the work of adhesion (*W*) between a solid and water can be divided into two interaction components. These represent dispersive and nondispersive interactions^25^ and can be written as
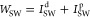
7and
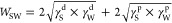
8where the dispersive component for water (γ_W_^d^) has a value of 21.8 and the polar component for water (γ_W_^p^) has a value of 51.0.^25^ The geometric mean expression used for *I*_SW_^d^ above is based on the work of Fowkes, while that for *I*_SW_^p^ is an extended one.

The surface wettability of each material was assessed by performing contact angle measurements in air (Kyowa Interface Science, DropMaster DM-500) with distilled, deionized water (Merck Millipore, Direct-QUV). These measurements were performed using as-polarized samples and samples 1 month after the polarization treatment. The contact angles were calculated using Young’s equation
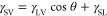
9where the subscripts S, L, and V refer to the solid, liquid, and vapor phases, respectively.

### Human Osteoclast Cultures

Peripheral mononuclear blood cells (PBMCs) were used as precursors of osteoclasts. The PBMCs were obtained from fresh human blood donated by healthy male according to the donation protocol approved by the Human Subjects Committee of the University of Turku and Tokyo Medical and Dental University as previously described.^3,26^ Blood was collected into the tubes with heparin as an anticoagulation factor. The anticoagulated blood was diluted 1:1 (v/v) with phosphate buffered saline (PBS), layered over the Ficoll–Paque Plus solution (Amersham Pharmacia Biotech, Uppsala, Sweden), and centrifuged at 1500 rpm for 15 min. The buffy coats were collected and washed twice with PBS and resuspended in a cell culture medium (α-MEM) containing 10% FBS, 1000 U/mL penicillin–streptomycin. The cells were placed onto the specimens at a density of 1 × 10^6^ cells/cm^2^ and cultured in the cell culture medium with an addition of 20 ng/mL RANKL (Peprotech 310-01) and 10 ng/mL M-CSF (R&D, 216-MC) for 14 days. Half of the media in each sample were changed every 3 to 4 days.

### Staining

The differentiation of the PBMCs into osteoclasts was confirmed by TRAP staining. The cells adhering to the specimens were washed with PBS twice, fixed with 4% paraformaldehyde in PBS for 20 min, and stained for TRAP (Sigma, 387A). The average number of TRAP-positive multinucleated (more than three nuclei) cells was calculated by a total of at least 30 fields on each specimen.

Cell morphologies were visualized by immunohistochemical staining. The cells adhering to the specimens were washed with PBS twice and fixed with 4% paraformaldehyde in PBS for 20 min. The cells were incubated with the blocking solution of 5% goat serum in 0.1% Triton X-100-PBS for 1 h, a mouse monoclonal anti-vinculin antibody in the blocking solution for 1 h at RT, Alexa-conjugated goat anti-mouse immunoglobulin in a blocking solution containing rhodamine phalloidin for 1 h at RT, and Hoechst staining solution. The cell morphologies were observed using a fluorescence microscope (Olympus IX71).

The quantification of the adhesion of osteoclasts was performed by the measurements of the diameters and thicknesses of the actin rings using NIH image. In addition, the quantification of the cell fusion was performed by counting the number of nuclei per cell. The average values of the measurements were calculated by a total of a minimum of 50 cells on each specimen.

### Analysis of Resorption Pits

After the specimens were examined using a microscope, the osteoclasts were removed from the specimens by scrubbing with a brush, following which each sample was washed with distilled water and then dried in air. The dried specimens were assessed with a three-dimensional color laser microscope (OLS4100, Olympus). The quantification of osteoclast resorption was performed by the measurements of the depth of each pit. The average values of the measurements were calculated by a total of at least 30 resorption pits on each specimen.

### Statistical Analysis

Accurate quantifications of the different samples were achieved by performing more than three independent experiments. Statistical analysis between groups was performed by an analysis of variance (ANOVA) with the Tukey formula for post hoc multiple comparisons, using the SPSS software package (version 22, Chicago, IL). A statistical significance level of *p* < 0.05 was used for all tests. All data are expressed herein as mean ± standard deviation (SD).

## Results

**Figure 1:**
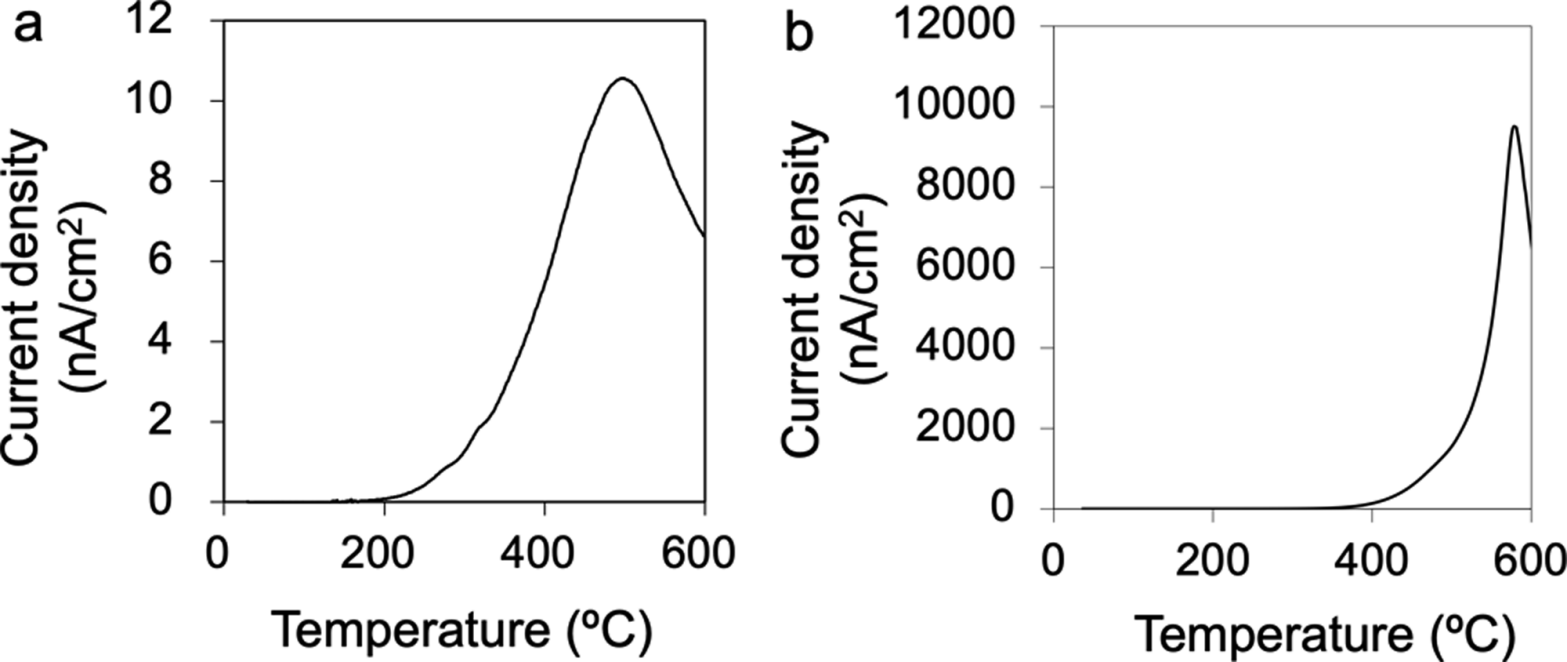
Typical TSDC curves obtained from (a) HA and (b) CA samples after polarization treatments.

Typical TSDC patterns for the polarized HA and CA are shown in Figure 1a,b, respectively. The TSDC curves for the HA and CA, respectively, increased at approximately 200 and 400 °C, reached maxima at approximately 510 and 580 °C, and then gradually decreased. The maximum current density was approximately 10 nA/cm^2^ for HA and 10,000 nA/cm^2^ for CA. The *Q* calculated from the TSDC data were 26 μC/cm^2^ for HA and 13 mC/cm^2^ for CA. The *E*_a_ obtained from the curves were 0.9 and 1.2 eV for HA and CA, respectively, while the τ at 37 °C were calculated to be 1 × 10^3^ and 2 × 10^15^ years. These differences in the stored charges, activation energies, and half periods are attributed to the different charged carrier particles participating in the polarization process. These comprised protons for HA and oxygen ions for CA.^27^

**Figure 2:**
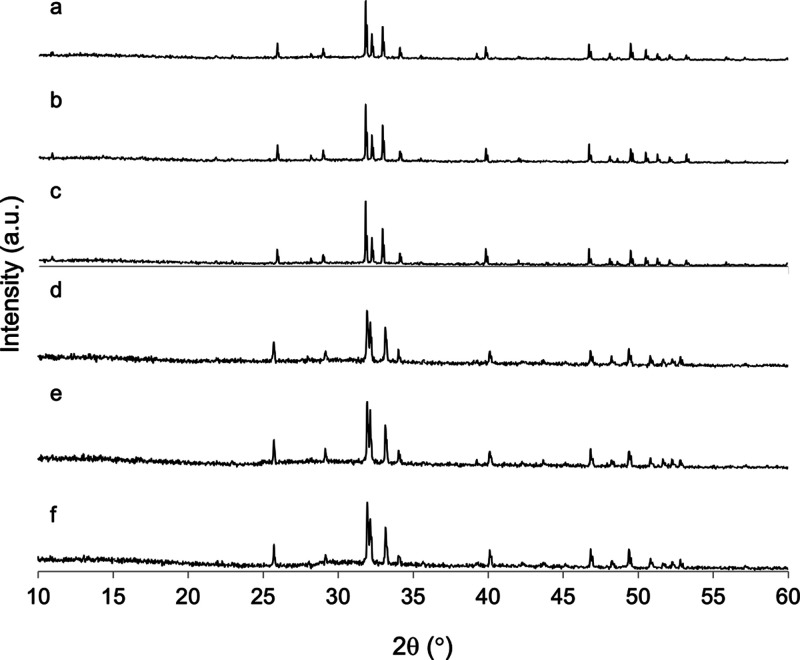
XRD patterns obtained from (a) standard HA, (b) HA-N, (c) HA-P, (d) standard CA, (e) CA-N, and (f) CA-P. Note that each one is extremely similar to that of single-phase hexagonal HA.

**Figure 3:**
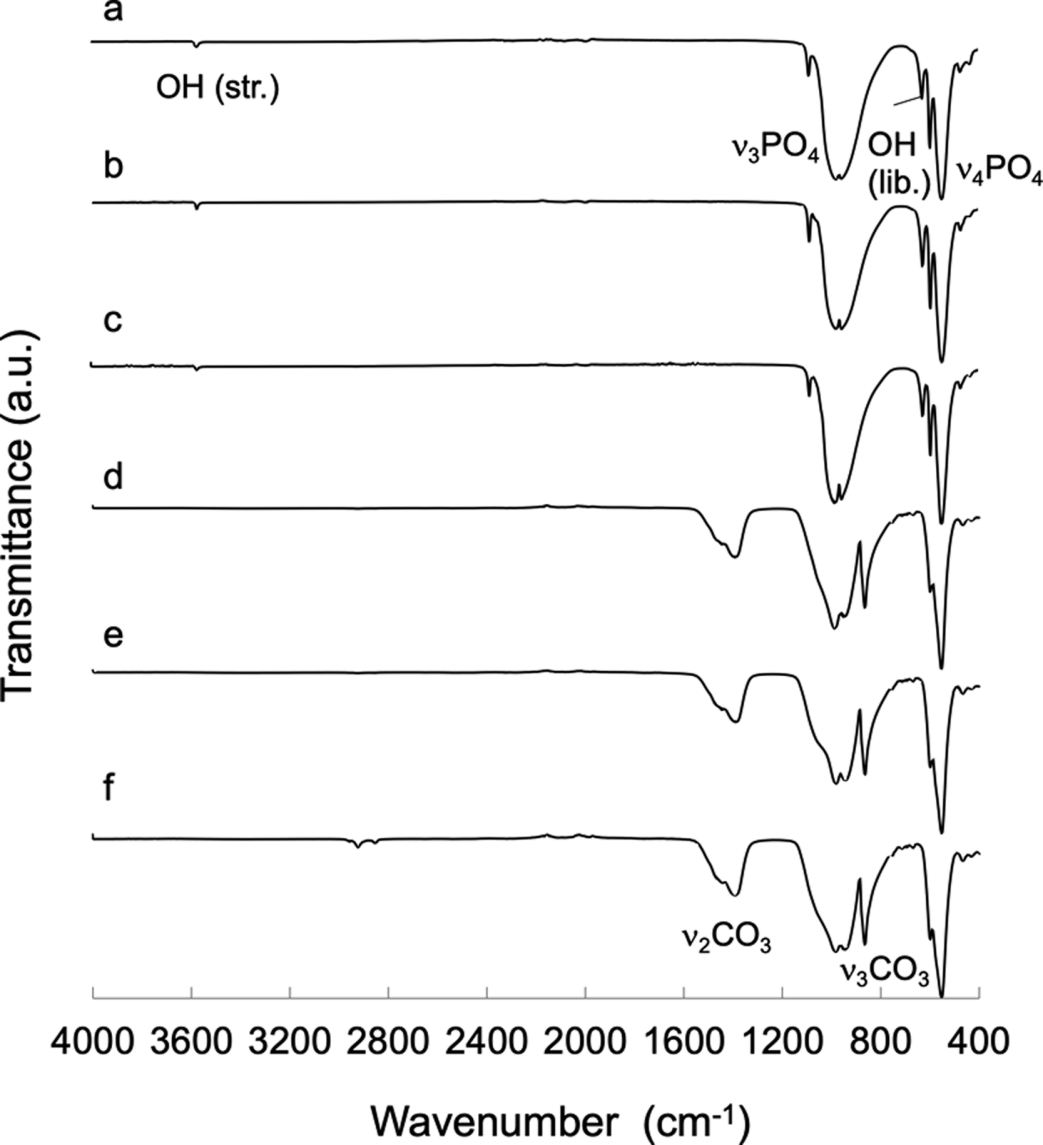
ATR-FTIR spectra obtained from (a) standard HA, (b) HA-N, (c) HA-P, (d) standard CA, (e) CA-N, and (f) CA-P.

The XRD patterns obtained from the HA and CA samples both with and without the polarization treatment were assigned to the HA standard pattern (ICDD no.9-432), meaning that each surface consisted of a single hexagonal HA phase (Figure 2). However, the peak related to the (002) diffraction for CA appeared at a lower angle than that for HA, suggesting B-type CA (in which carbonate ions are substituted for phosphate sites in the HA lattice).^12^ The ATR–FTIR spectra of all specimens contained peaks assigned to phosphate ions at 1045, 1089, 601, 575, and 567 cm^–1^ (Figure 3). The spectra obtained from HA contained stretching and bending vibrations of hydroxide ions at 3570 and 630 cm^–1^, respectively. The spectra obtained from CA also contained carbonate peaks at 1415, 1450, and 871 cm^–1^. These data confirm that the sintered CA specimens used in this study comprised B-type CA containing approximately 8 wt % carbonate ions substituted at phosphate sites in the apatite crystal lattice.^17^

**Figure 4:**
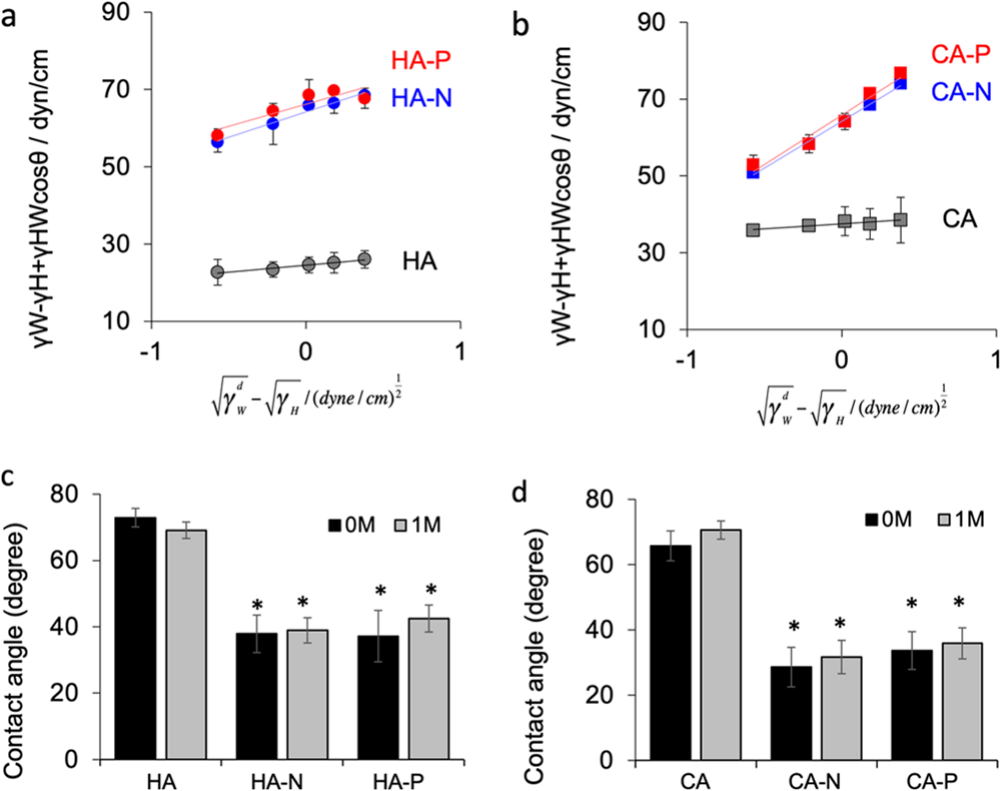
Contact angles determined using the two-phase liquid method in hexane, heptane, octane, decane, and hexadecane with distilled, deionized water for (a) HA and (b) CA with and without polarization treatments. Black, blue, and red symbols indicate standard, negatively charged, and positively charged surfaces, respectively. Contact angle data for (c) HA and (d) CA with and without polarization treatments determined using distilled, deionized water in the as-polarized state and 1 month after the polarization treatments. The HA and CA surfaces presented higher angles, indicating that these materials were more hydrophobic.

The contact angles in hydrocarbon oils determined for HA and CA specimens with and without polarization using the dual liquid method are presented in Figure 4a. The dispersive and polar components of the surface free energy values were calculated from the slopes and *y* intercepts of these plots, and the surface free energies were obtained by summing the dispersion and polar components (Table 1). The resulting surface energies were 38.6 mJ/m^2^ for standard HA and 51.1 mJ/m^2^ for standard CA. The surface energy values for initial polarized HA and CA were found to be increased by factors of approximately 1.7 and 1.5 times, respectively, relative to the unpolarized samples. After 1 month, these standard values for HA and CA were found to increase to 40.3 and 55.0 mJ/m^2^, respectively. In addition, the surface energy values for the polarized HA and CA after 1 month were found to be increased by factors of approximately 1.7 and 1.5 times, respectively, relative to those of the unpolarized 1 month-aged samples.

**Table 1:** Surface Free Energies of HA and CA Samples with and without Polarization Treatments as Calculated Using Jouany’s Eequation^a^

	as-polarized			after 1 month		
	dispersive components: γ_S_^d^ (mJ/m^2^)	polar components: γ_S_^p^ (mJ/m^2^)	surface free energy (mJ/m^2^)	dispersive components: γ_S_^d^ (mJ/m^2^)	polar components: γ_S_^p^ (mJ/m^2^)	surface free energy (mJ/m^2^)
HA	2.23	36.4	38.6	2.2	38.1	40.3
HA-N	11.8	55.4	67.2	17.0	51.6	68.6
HA-P	13.3	54.8	68.1	11.0	53.6	64.6
CA	6.10	45.0	51.1	9.84	45.2	55.0
CA-N	17.7	62.0	79.7	17.6	55.3	72.9
CA-P	13.6	61.2	74.8	14.8	57.3	72.1

a The dispersion and polar components of the surface free energies were calculated based on the slopes and *y* intercepts of plots of contact angles determined in hydrocarbon oils (Figure 4a,b). The surface free energy values were calculated by summing the dispersion and polar components.

The contact angle values determined in air using distilled, deionized water for HA and CA were significantly decreased after the electrical polarization treatment (Figure 4c,d). The angles were 73 ± 2.8° for the standard HA and 66 ± 5.1° for the standard CA prior to polarization while the values were 37 ± 4.9°, 33 ± 5.6°, 34 ± 5.8°, and 29 ± 6.1° for the HA-N, HA-P, CA-N, and CA-P, respectively, after polarization. The surfaces of the polarized HA and CA therefore exhibited lower contact angles, meaning that the wettability of each negative and positive surface was improved. The contact angle values were remeasured 1 month after the polarization treatment and compared with those of the as-polarized surfaces and no significant differences were found. Thus, the increased surface free energy and improved surface wettability were maintained for at least this length of time.

**Figure 5:**
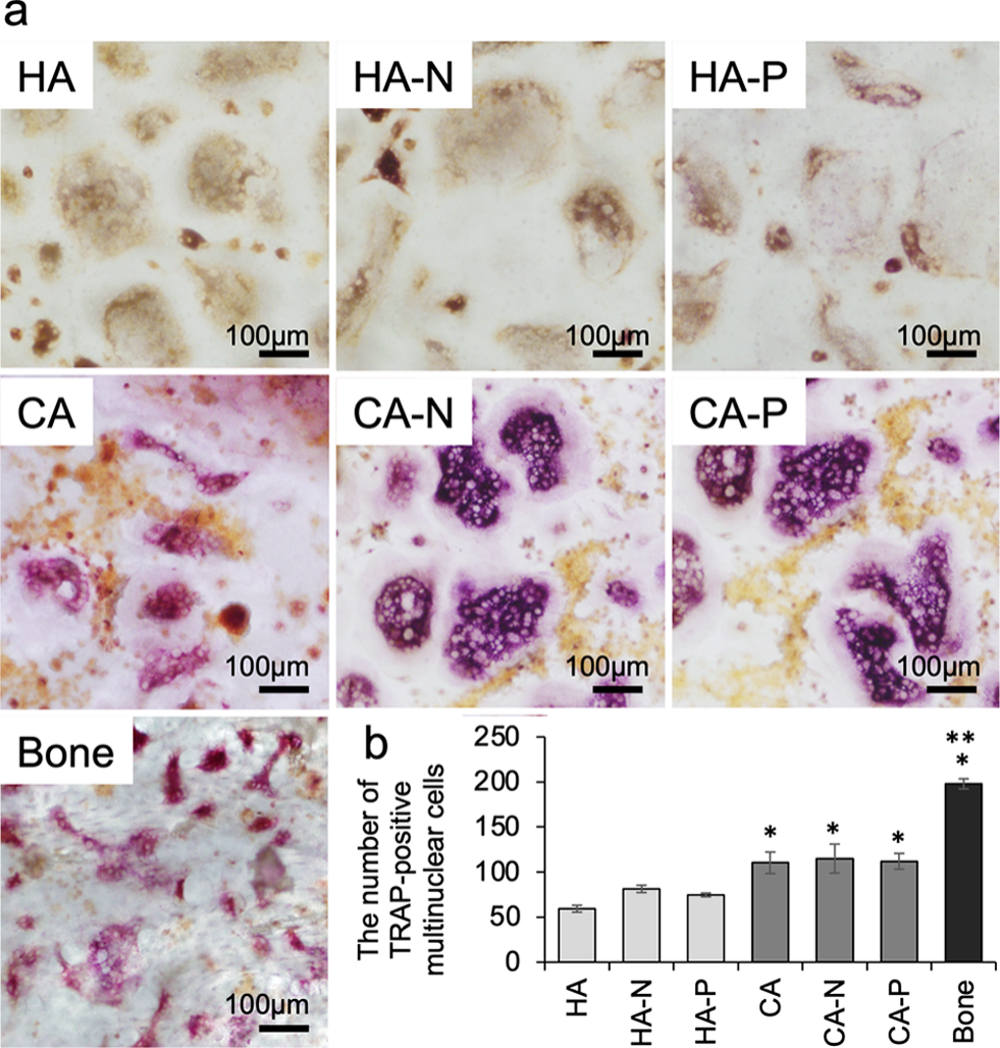
(a) TRAP staining of the cells cultured on bone slices and on HA and CA samples with and without polarization treatments. The bars indicate 100 μm. (b) Quantities of TRAP-positive multinucleated cells used to quantify the differentiation of PMBCs into osteoclast precursors or osteoclasts on seven specimens. The number of TRAP-positive multinucleated cells was significantly increased on the CA samples and bone slices compared to the HA samples. *p < 0.002 and **p < 0.001 compared with the others. Error bars indicate ± one standard deviation.

Osteoclasts derived from human PBMCs were positively stained for TRAP on the surfaces of all specimens after culturing with osteoclast differentiation factors for 14 days (Figure 5a). The TRAP staining showed that the PBMCs adhering to the specimens had differentiated into osteoclasts. Some TRAP-positive cells adhering to the specimens were small and mononuclear, suggesting that they had not completely differentiated into mature osteoclasts. The quantities of multinuclear TRAP-positive cells (which indicate osteoclast differentiation) were significantly larger on the CA samples compared with the numbers on the HA samples (Figure 5b), while the bone slices exhibited the highest concentration of multinuclear TRAP-positive cells.

**Figure 6:**
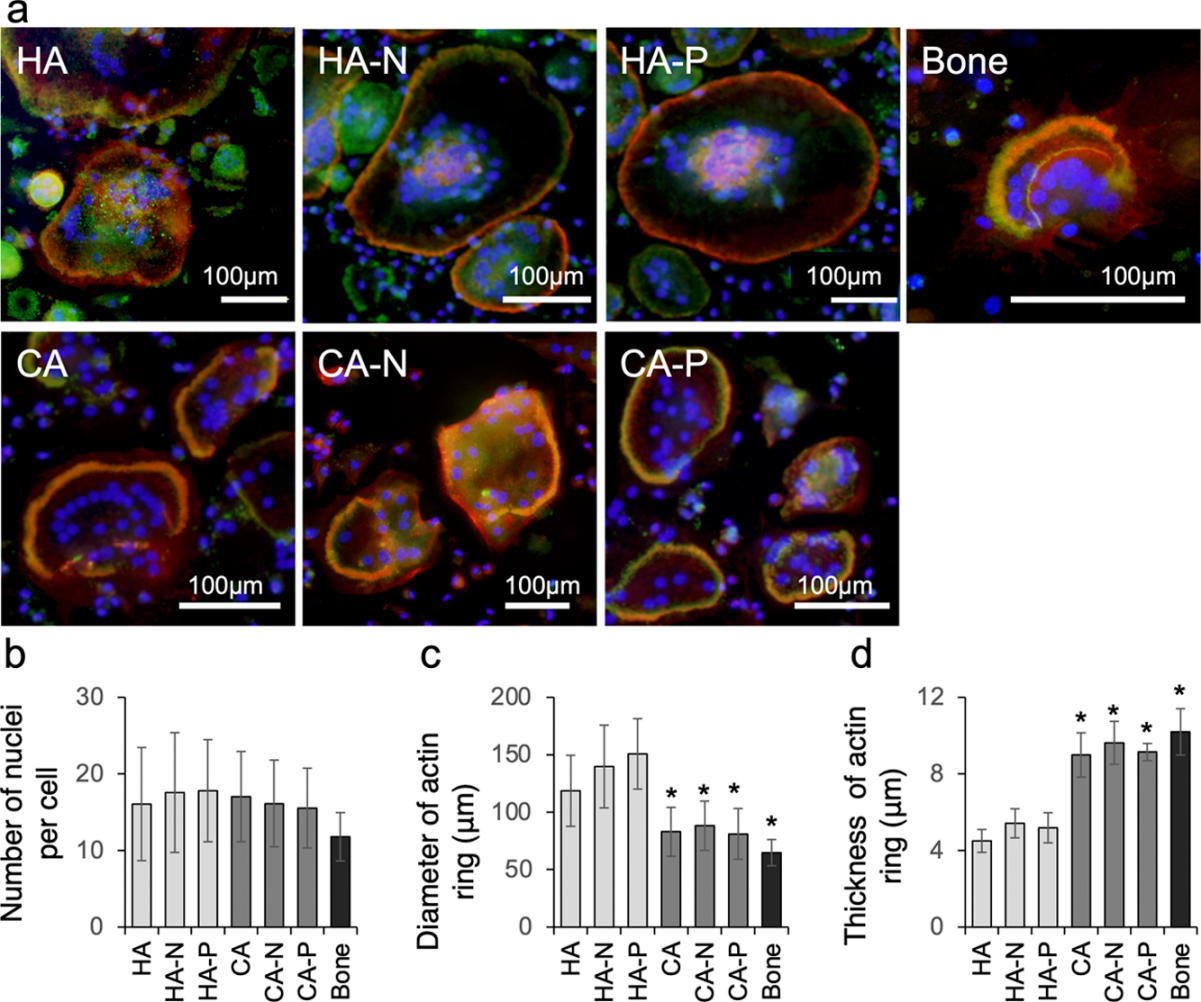
(a) Morphologies of the osteoclasts adhering to bone slices and to biomaterial samples with and without polarization treatments, as visualized by the fluorescent staining of nuclei (blue), vinculin (green), and actin filaments (red). Actin ring structures were formed in the osteoclasts on each surface, and the vinculin molecules were co-localized with the actin rings in the osteoclasts cultured on the bone slices and CA specimens, while the vinculin molecules were scattered throughout the central regions of the cells on the HA samples. The bars indicate 100 μm. (b) Quantities of nuclei in each osteoclast as a means of quantifying the differences in the osteoclast morphology. The number of nuclei in each cell was essentially equivalent for all specimens. (c) Diameters of actin rings in osteoclasts as a means of quantifying the differences in the actin ring morphologies. The actin rings in the osteoclasts cultured on the CA samples and bone slices were significantly smaller than those on the HA specimens. (d) Thicknesses of actin rings in osteoclasts as a means of quantifying the differences in actin ring morphologies. The rings of osteoclasts cultured on the HA specimens were thinner, whereas those of the osteoclasts cultured on the CA specimens were thicker and similar to those observed on the bone slices. *p < 0.001 compared with the others. Error bars indicate ± one standard deviation.

Figure 6 shows the results of vinculin (green), actin (red), and nuclei (blue) labeling of the adhered osteoclasts (Figure 6a). Actin-based sealing rings as a marker for active bone resorbing were observed in the osteoclasts on the HA, CA, and bone slices. The osteoclast morphologies were quantified by assessing the number of nuclei in each cell, the thicknesses of the actin rings, and the diameters of the actin rings. The number of nuclei in each cell was essentially equivalent for all specimens, suggesting that the biomaterials did not affect cell fusion during the osteoclast differentiation process (Figure 6b).

Three differences were identified in the fluorescence images obtained from the HA and CA specimens, one of which was the vinculin-immunoreactive distribution, that is, one of the cytoskeletal molecules at the focal adhesions in the sealing zone of resorbing osteoclasts (Figure 6a). Specifically, vinculin molecules were co-localized with the actin rings of the osteoclasts cultured on the CA and bone slices but scattered throughout the central regions of the osteoclasts cultured on the HA. The second difference was in the actin ring sizes of the osteoclasts (Figure 6c). The ring sizes of osteoclasts cultured on the CA and bone slices were significantly smaller than those on the HA. These larger actin rings on HA indicated that the osteoclasts spread widely compared to the CA and bone slices. The third difference was the thickness of the osteoclast actin rings (Figure 6d). The rings of osteoclasts cultured on the HA specimens were thinner, whereas those of the osteoclasts cultured on the CA specimens were thicker and similar to those observed on the bone slices.

**Figure 7:**
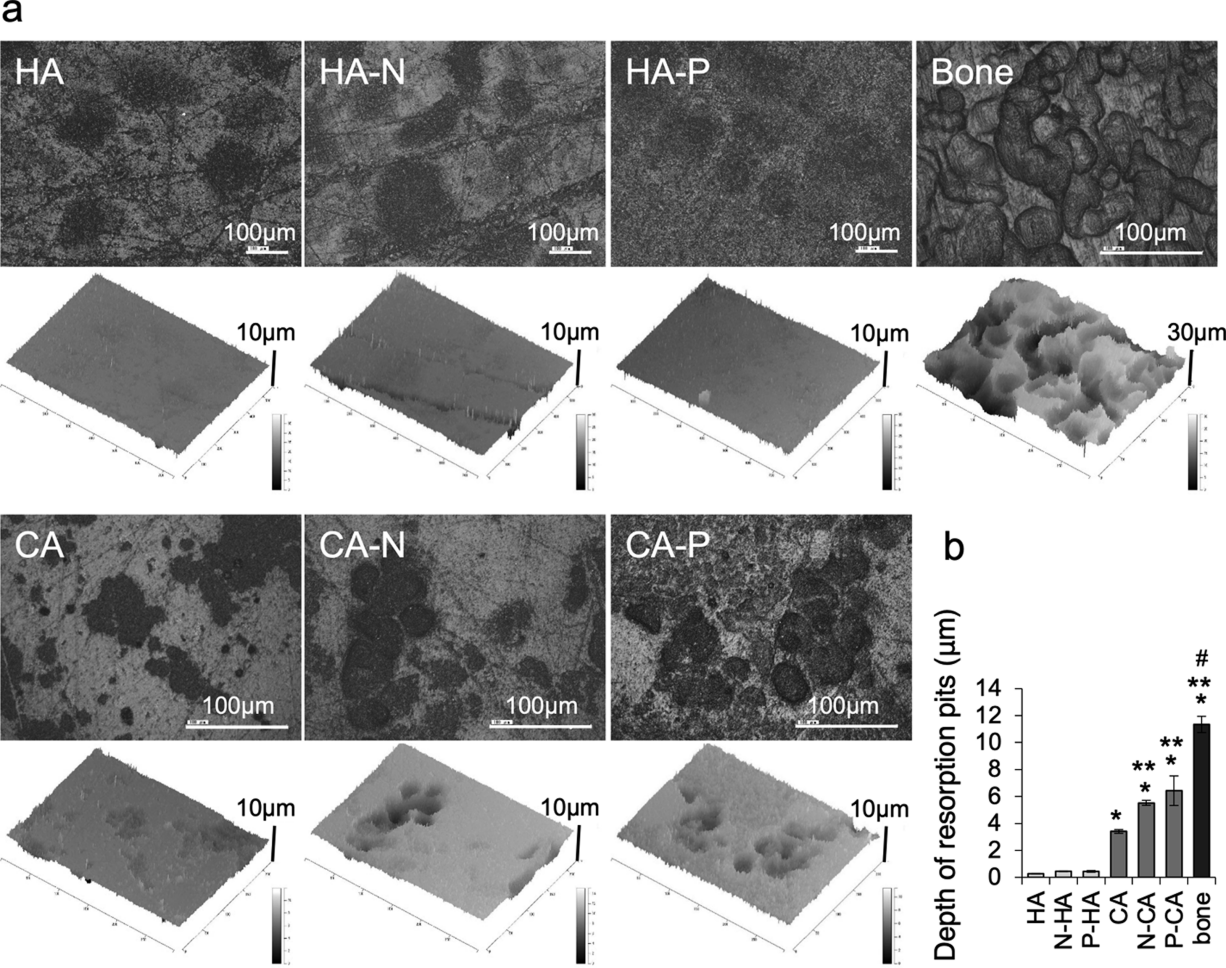
(a) Images of the resorption pits formed on bone slices and on biomaterial samples with and without polarization treatments. The bars indicate 100 μm. (b) Depths of the surfaces after osteoclast culturing and cell scrubbing as a means of quantifying the differences in resorption pits. The osteoclasts formed deeper resorption pits on the bone slices than those on the other specimens. The pits on the CA samples were larger than those on the HA samples. The pits on the polarized CA were also deeper than those on the standard CA surface. *p < 0.001, **p < 0.002, and #*p* < 0.001 compared with the others. Error bars indicate ± one standard deviation.

Resorption pits were observed on all sample surfaces using three-dimensional laser microscopy (Figure 7a). The distinct pits were formed on the bone slices and CA samples, whereas ambiguous, shallow pits appeared on the HA sample. The measurements of the depth of the resorption pits revealed that the osteoclasts resorbed the CA samples approximately 11 times deeper than those on the HA samples (Figure 7b). The pit depths on the CA-N and CA-P samples were approximately 18 and 21 times greater than those on the standard HA sample and 1.6 and 1.9 times greater than those on the standard CA sample, respectively.

## Discussion

The results of this work show that the carbonate ions substituted in an apatite crystal structure affects the osteoclast differentiation and resorption and that polarization modifies the resorption of human osteoclasts. Here, we discuss the effects of the carbonate ions and surface free energy in relation to the conventional understanding of surface characteristics and cell activities.

The trials involving osteoclast cultures showed that the human PBMCs differentiated into mature osteoclasts and were activated to resorb the substrate when using the CA samples. These results are in agreement with our previous study,^3,17^ in which standard CA was found to enhance osteoclast differentiation, as indicated by increases in the number of TRAP-positive multinucleated cells. This prior work also indicated the formation of thick, compact actin rings that create tight seals between the osteoclasts and the CA, as well as the formation of deeper resorption pits compared with those formed on standard HA. The surfaces of CA specimens were characterized as a single, highly crystalline HA phase and that phosphate ions were partially substituted for carbonate ions, meaning that the material could be classified as B-type CA^17^ and had properties similar to bone. It should be noted that the mineral component of a bone matrix is nonstoichiometric HA with a partial substitution of carbonate, potassium, magnesium, chloride, and sodium ions within its crystal structure.^4^ Therefore, one possible explanation for the variations in osteoclast differentiation and resorption is that changes in the material properties with the substitution of carbonate ions into the HA crystal structure increased the similarity to the bone tissue.

Osteoclast activity is an essential aspect of controlling bone remodeling and is related to the organization of the actin cytoskeleton to form a sealing zone that anchors the osteoclasts to the bone matrix. Some of the parameters of material characterization such as surface roughness,^13,14^ crystallinity of the solid surface,^15^ solubility,^28^ and surface free energy^29^ affects the morphology of the actin rings in osteoclasts. In the present study, the synthesized substrates had approximately equivalent surface roughness values as a result of polishing. In addition, the XRD data showed that the material surfaces consisted of a single highly crystalline HA phase. Therefore, the solubilities and surface energies of the synthesized specimens are believed to have had the greatest effects on osteoclast resorption.

The higher solubility of CA would be expected to promote osteoclast resorption by calcium and phosphate ions released from the bone mineral in the extracellular matrix at the sealing zone between the bone matrix and osteoclasts. The released ions would be expected to stimulate the surrounding osteoclasts to resorb the bone matrix. Calcium and phosphate ions are released from the bone matrix during osteoclast resorption, and this release stimulates the resorption of the surrounding osteoclasts. The sensitivity of osteoclasts to extracellular calcium concentrations is reported to vary throughout the osteoclast activation process, which comprises resting, migrating, and resorbing.^30^ Osteoclasts are not sensitive to increase extracellular calcium concentrations during the resorbing process, while osteoclasts are more sensitive during the resting process. Specifically, resting osteoclasts surrounding the resorbing osteoclasts are stimulated by Ca^2+^ released from the bone mineral in the extracellular matrix and subsequently differentiate to resorb the intact areas of the bone extracellular matrix. The calcium ions^31^ and phosphate ions^32^ will be released from CA resorbed by the activated osteoclasts into the extracellular medium for cell culture. These ions then stimulate and differentiate the surrounding resting osteoclasts such that the intact CA surface is resorbed.

Another possible explanation for the observed osteoclast formation on the CA is acidification resulting from the release of bicarbonate or carbon dioxide from the CA surface. There are potential causes of local acidosis as a result of inflammation,^33^ fractures,^34^ and tumors.^35^ Under the local acidic conditions, such as in the case of surgical wounds or fractures, bicarbonate or carbon dioxide could possibly be released along with phosphate and calcium ions. The acidic conditions at these local sites are known to induce osteoclast resorption.^36^ In the present study, the osteoclasts displayed a more spread cell morphology on the HA samples compared to that on the CA specimens (Figure 6a,c) and formed deeper resorption pits on the surfaces of the CA materials (Figure 7). These results can possibly be attributed to the higher solubility of CA because the standard CA exhibited increased solubility compared to the standard HA.^17^

The surface energy value is known to affect the initial adherence, spread, and formation of the collagen fibrils of human osteoblast-like cells^29^ as well as the cellular morphology of human osteoclasts.^2^ Human osteoclasts displayed a more spread cell morphology on A-type CA (in which carbonate ions are substituted for hydroxyl sites in the HA crystal structure).^2^ Redey et al. have suggested that the different cell morphologies on A-type CA results from a decrease in the polar component of the surface free energy compared with HA. Such reports suggest that the surface free energy affects cell activities on CA. Although the ion substitution sites in the crystal structures were different between A-type CA and B-type CA, the B-type CA showed an increase in surface energy by a factor of approximately 1.5 following polarization (Figure 4). In addition, the polarization treatment enhanced osteoclast resorption on the CA (Figure 7). Overall, these results indicate that increases in the surface free energy are one of the important factors in the enhancement of osteoclast resorption on the polarized CA.

Another possible explanation for the enhanced osteoclast resorption on the polarized CA is based on the changes in the protein conformation. Many different proteins that are involved in cell adhesion were included in the cell culture medium, and increasing the surface free energy could have changed the protein conformation related to the adhesion of osteoclasts. Each CA specimen was immersed in the cell culture medium prior to osteoclast culturing and so was presumably covered with a similar amount of the adsorbed proteins. Taking into account both the evident changes in surface free energy caused by ion substitution in the apatite crystal structure^29^ and variations in the conformations of adhesion proteins as a consequence of surface chemistry,^37,38^ it is possible that the protein conformation on the substrates varied as a consequence of differences in the surface free energy values of the samples. Variations in the exposed domains of the proteins adsorbed on the samples could possibly be lead from the differences in the conformation of the adhesion proteins.

During bone remodeling, the osteoclast resorption period is known to be 30 to 40 days and is followed by bone formation over a period of 150 days.^39^ In the work reported herein, the polarization effects were evidently maintained over at least 1 month because the increased surface free energy and improved surface wettability were retained over this time span (Table 1 and Figure 4c,d) and because the surface charges on the HA induced by polarization were maintained during sterilization and cell culture.^40^ Considering that the τ values at 37 °C obtained from the TSDC curves were 1 × 10^3^ years for HA and 2 × 10^15^ years for CA (Figure 1), the surface charges would be expected to remain throughout the bone remodeling period. This stability of the surface charges indicates that electrical stimulation and the concurrent polarization could be beneficial as a clinical treatment to accelerate bone remodeling. The results of this work should contribute to the future design of cell-mediated bioresorbable biomaterials capable of resorption by osteoclasts and of serving as scaffolds for bone regeneration.

## Conclusions

In the present study, novel and important information regarding the surface characteristics of polarized inorganic biomaterials and the behavior of osteoclasts on these materials was provided. Polarization was found to improve the surface wettability of HA and CA as a result of increases in surface free energy, and this effect was still present after 1 month. In addition, trials in which osteoclasts were cultured on various substrates showed that polarized CA enhanced osteoclast resorption but did not affect the TRAP staining and morphology of osteoclasts.
